# Prevention of Prostate Tumor Development by Stimulation of Antitumor Immunity Using a Standardized Herbal Extract (Deep Immune®) in TRAMP Mice

**DOI:** 10.1155/2018/9707543

**Published:** 2018-05-14

**Authors:** Peihe Liang, Jia Guo, Shadan Li, Qiunong Guan, Terry Vanderheyden, Alan So, Yuzhuo Wang, Tao Chen, Caigan Du

**Affiliations:** ^1^Department of Urologic Sciences, The University of British Columbia, Vancouver, BC, Canada; ^2^Department of Urology, The Second Affiliated Hospital, Chongqing Medical University, Chongqing, China; ^3^Department of Urology, Renmin Hospital of Wuhan University, Wuhan, Hubei, China; ^4^Department of Urology, Chengdu Military General Hospital, Chengdu, Sichuan, China; ^5^St. Francis Herb Farm Inc., Combermere, ON, Canada; ^6^Vancouver Prostate Centre, Vancouver, BC, Canada; ^7^BC Cancer Agency, Vancouver, BC, Canada; ^8^Key Laboratory of South Subtropical Plant Diversity, Fairy Lake Botanical Garden, Shenzhen & Chinese Academy of Sciences, Shenzhen, Guangdong, China

## Abstract

Low-risk prostate cancer (PCa) does not require immediate treatment, but PCa progression after years of active surveillance will need the treatment. This study was to test the efficacy of immunostimulant Deep Immune (DI) in controlling PCa progression. DI is an extract of eight different medicinal herbs.* In vitro* activity of DI was determined by phagocytosis activation using flow cytometric analysis of fluorescence-labeled latex bead uptake, expression of immune-modulating 84 genes using PCRarray, and tumor killing using coculturing with immune cells. Anti-PCa activity of DI* in vivo* was examined in male TRAMP mice.* In vitro* DI stimulated phagocytosis and expression of a panel of inflammatory mediators (C4b, CXCL3, lymphotoxin, NOS2, TLR1, TNF, and TNFSF14) in cultured macrophages and increased tumor killing of both macrophages and TRAMP mouse splenocytes. Daily intake of this herbal product significantly suppressed the tumor size (*P* = 0.0368) with lower histopathologic scores (*P* = 0.0364) in TRAMP mice, which were associated with an increase in both splenocyte cytotoxicity against tumor cells and numbers of CD8 T cells, macrophages, and dendritic cells in the spleens* in vivo*. In conclusion, daily intake of DI prevents PCa progression in TRAMP mice, suggesting the possible effectiveness of the immunostimulant herbal products on prevention of PCa progression after diagnosis of low-risk PCa.

## 1. Introduction

In developed countries such as the United States of America and Canada, prostate cancer (PCa) is the most common cancer in men and is one of the leading causes of cancer death [1, 2]. Once localized low-risk PCa (PSA: ≤10 ng/mL; Gleason score: ≤6; clinical stage: T1-2a) is diagnosed, the primary option of guideline-recommended treatments is active surveillance (AS), because in most cases of newly diagnosed, low-risk PCa, tumor cells in the prostate gland grow slowly and do not necessarily need to be treated immediately with a definitive therapy of curative intent [3–5]. However, PCa in these patients can slowly progress to high-risk aggressive cancer and eventually a definitive therapy is required. Indeed, many cohort studies have reported that 30–49% of men who initially were on AS end up being treated for their PCa during various years of follow-up (mean: 4.33–7.7 years) [6–8]. Therefore, in order to avoid the risk of significant side effects of definitive treatments (mostly radiation) and to improve quality of life for those who only have non-life-threatening PCa at the beginning, a feasible, relatively cost-effective, and therapeutically effective method of controlling the progression of this low-risk PCa is needed.

It has been documented for some time that immune cells can recognize and eliminate tumor cells via tumor immunosurveillance, an important host protection process of maintaining regular cellular homeostasis against tumor development [9]. Thus, by activation of this specific antitumor immunity, tumor development can be controlled or even eliminated. Indeed, in the treatment of PCa, a large, phase III trial has shown a survival improvement of more than 4 months in PCa patients by an active systemic immunotherapy using sipuleucel-T (Provenge®) [10, 11], suggesting that stimulation of natural antitumor immunity in our body may be a feasible strategy to control prostate tumor development. Based on the Canada Health Measures Survey (2007/2009), 43% of adults (18–79 years old) regularly use natural health products (NHPs) [12], implying that using NHP as a control strategy for PCa progression is acceptable and practicable in patients with low-risk PCa. The Deep Immune (DI) is produced as a commercial NHP product by the extraction of eight different medicinal herbs (Table 1) in St. Francis Herb Farm® Inc. (Combermere, ON, Canada) following government regulations. Literature search shows that the extracts from these herbs have been demonstrated to have immunostimulatory activities* in vitro* and/or* in vivo* [13–20], but whether or not this immunostimulant Chinese herbal formulation can be used for controlling PCa progression has not yet been investigated.

A transgenic strain of mice with adenocarcinoma of the mouse prostate (TRAMP) was created more than 20 years ago [21], and PCa in these TRAMP mice closely mimics human PCa with respect to progression, androgen independence, biochemistry, and pathology [22, 23]. The objective of this preliminary study was to evaluate the efficacy of the DI NHP as a prevention strategy in an attempt to control PCa progression in TRAMP mice.

## 2. Materials and Methods

### 2.1. Plant Materials and Cell Culture

Liquid DI was provided by St. Francis Herb Farm Inc. (Canada) and is a standardized herbal extract product prepared by the manufacturer using eight different medicinal herbs (Table 1). For this experimental study, this product was further dried by lyophilization and reconstituted with an appropriate solution for both chemical and biological measurements.

TRAMP-C2 cells, a murine prostate tumor cell line derived from TRAMP mice [24], were purchased from the American Type Culture Collection (ATCC, Manassas, VA, USA) and were maintained in Dulbecco's modified Eagle's Medium (DMEM) complete medium (Invitrogen Canada, Burlington, ON, Canada), supplemented with 5 *μ*g/mL bovine insulin, 10 nM dehydroisoandrosterone, and 10% fetal bovine serum (FBS). RAW 264.7 cells, a murine macrophage-like cell line, were purchased from ATCC and were grown in DMEM complete medium supplemented with 10% FBS. Single cell splenocytes were prepared from the spleens of TRAMP mice, as described previously [25], and were suspended or incubated in Roswell Park Memorial Institute (RPMI) 1640 complete medium (Invitrogen Canada) containing 10% FBS. All cells were grown at 37°C in a humidified 5% CO_2_/95% air incubator.

### 2.2. Chemical Composition Analyses Using a Spectrophotometer

The composition of DI (sugar, triterpenoids, flavonoids, and protein) was determined by using spectrophotometric methods. Total sugar including polysaccharides and monosaccharides was determined by the standard phenol sulfuric acid method using glucose as a reference standard as described previously [26], triterpenoids saponins by the vanillin-sulfuric acid method using Quillaja saponin as a reference [27], flavonoids by the aluminium chloride colorimetric method using quercetin as a reference [28], and protein by the Bio-Rad protein assay following the manufacturer's protocol (Bio-Rad Lab, Hercules, CA, USA).

### 2.3. Procedure and Conditions of Ultra Performance Liquid Chromatography-Quadrupole-Time-of-Flight Mass Spectrometer (UPLC-Qtof-MS) Analysis

#### 2.3.1. Extraction Procedure

According to UPLC-Qtof-MS operations manual (protocol), five mg of dried DI product was dissolved in 1 mL of 30% methanol, followed by centrifugation at 8,000 ×g for 5 min. The supernatant was collected for chemical analysis using UPLC-Qtof-MS system.

#### 2.3.2. UPLC Condition

UPLC was performed with a Waters ACQUITY Xevo G2 system (Waters Co., MA, USA), equipped with a binary solvent delivery system, autosampler. The chromatography was performed on a Waters ACQUITY UPLC® BEH C18 column (2.1 mm × 50 mm, 1.7 *μ*m). The mobile phase consisted of 0.1% formic acid in water (A solution) and 0.1% formic acid in acetonitrile (B solution). The UPLC elution condition was optimized as follows: 0 min: 90% of A and 10% of B; 0–18 min: 85–35% of A and 15–65% of B; 18–20 min: 35–5% of A and 65–95% of B; 20–23 min: 5% of A and 95% of B. The flow rate was at 0.30 mL/min and the column and autosampler were maintained at 35°C and 10°C, respectively. The injection volume was 10 *μ*L.

#### 2.3.3. MS Condition

The Qtof-MS detection was performed on a Xevo G2 system (Waters Co., Manchester, UK). The data acquisition mode was MS^E^. The experiment was performed on both ESI^+^ and ESI^−^ ionization modes with data acquisition range from 100 to 1500 Da. The source temperature was 120°C, and the desolvation temperature at 350°C with desolvation gas flow at 900 L/h. The lock mass compound was leucine enkephalin (3 *μ*L/min) at a concentration 10 ng/mL for real-time recalibration of* m/z* shifts. The capillary voltage was 3 KV. The cone voltage was 40 V for ESI^−^ or 30 V for ESI^+^. The collision energy was 10 eV for low-energy scan and 30 eV for high-energy scan, respectively.

#### 2.3.4. Chemical Analysis by UNIFI Software

All the MS spectrometry data were further processed using UNIFI software (Waters Co.), and the identity of each peak in UPLC-Qtop MS base peak ion (BPI) chromatograms was based on the mass error and no of fragment matched in a Traditional Medicine library provided within the UNIFI informatics platform (Waters Co.).

### 2.4. Phagocytosis Assay

Phagocytosis of RAW264.7 cells was measured by using fluorescence-labeled latex beads (Sigma-Aldrich Canada, L-3030, carboxylate-modified, average size 2 *μ*m) as described previously [29]. In brief, RAW264.7 cells (0.1 × 10^6^ cells/0.5 mL/well) were seeded in the absence or presence of various concentrations (25–100 *μ*g/mL) of DI product in 24-well plates for overnight, followed by incubation with 2.5 *μ*L of fluorescent latex beads per well for 2 h. Phagocytosis was determined by the number of cells engulfing beads, which was measured by using a flow cytometer and analyzed and presented as the increased intensity of fluorescence (%) in phagocytic cells as compared to background controls (no beads) using FlowJo software (Tree Star Inc., Ashland, OR, USA).

### 2.5. PCR Array

The expression of 84 genes involved in “autoimmunity and inflammatory immune responses” in cultured RAW264.7 cells stimulated with 100 *μ*g/mL of herbal extract (based on the nitric oxide production, see below) compared to untreated controls (culture medium only) was quantitatively examined using PCR Arrays kit following manufacturer's instruction (SABiosciences, QIAGEN Inc., Valencia, CA, USA). After 24 h of stimulation with the herbal extract, the total RNA in each group was extracted from four separate treatments (*n* = 4) and purified using the RNeasy Microarray Tissue Mini kit (QIAGEN) and converted to cDNA using RT^2^ First Strand Kit (QIAGEN). The expression of selected genes was amplified by real-time PCR using RT^2^ Profile PCR arrays (QIAGEN). Data was analyzed using Web-based PCR Array Data Analysis Software (http://www.SABiosciences.com/pcrarraydataanalysis.php.).

### 2.6. Nitric Oxide (NO) Measurement

NO production in RAW264.7 macrophage cultures was determined after 24 h of stimulation with different concentrations of DI product (0, 25, 50, or 100 *μ*g/mL). Lipopolysaccharide (LPS, Sigma-Aldrich, Canada) at 1 *μ*g/mL was used as a positive control in this experiment. Because NO secreted from cells is rapidly oxidized to nitrite in the culture medium, nitrite concentrations in the medium were determined using the Griess method as a measurement of NO production. In brief, 50 *μ*L of culture supernatant was first mixed with 50 *μ*L of 1% sulfanilamide in 5% phosphoric acid in 96-well plates in triplicate and then was incubated for 10 min; then 50 *μ*L of 0.1% naphthylethylenediamine dihydrochloride in distilled water was added per well. The absorbance was read at 550 nm after incubation for 10 min, and the level of NO/nitrite was calculated using a standard curve with known sodium nitrite concentrations.

### 2.7. Nitric Oxide Synthase 2 Protein Expression

Cellular levels of nitric oxide synthase 2 (NOS 2 or iNOS) protein expression were examined by Western blot as described previously [29]. Briefly, total cellular protein was extracted from cultured RAW264.7 macrophages after 24 h of stimulation with 100 *μ*g/mL of herbal extract or medium only (Control), and approximately 100 *μ*g protein/sample was fractionated by 7% SDS-PAGE and consequently was transferred onto a nitrocellulose membrane. NOS 2 protein bands were identified with primary rabbit polyclonal anti-NOS 2 antibody (N-20) (Santa Cruz Biotech, Santa Cruz, CA, USA) and secondary goat anti-rabbit IgG antibody (Vector Lab., Burlingame, CA, USA). The NOS 2 protein-antibody complex was visualized by an enhanced chemiluminescence assay (ECL, Amersham Pharmacia Biotech, Buckinghamshire, England). Blots were reprobed using anti-*β*-actin IgG (Sigma-Aldrich Canada, Oakville, ON) for confirmation of loaded protein in each sample.

### 2.8. Calcein-Acetyoxymethyl (Calcein-AM) Cytotoxicity Assay

The lytic activity of RAW264.7 macrophages or effector splenocytes from TRAMP mice against target TRAMP-C2 cells was evaluated by Calcein-AM cytotoxicity assay using a coculture system as described previously [29]. Briefly, TRAMP-C2 cells (1 × 10^6^ cells/mL) were labeled with Calcein-AM dye (15 *μ*M) in a complete DMEM medium at 37°C for 30 min, followed by an extensive washing with the DMEM medium to remove extracellular dye. The fluorescent dye-labeled TRAMP-C2 cells (0.2 × 10^6^ cells/0.25 mL/well) were seeded with macrophages or splenocytes at different ratios in 24-well plates and were incubated at 37°C in 5% of CO_2_ for 24 hrs. Supernatants were collected after the plate was spun at 1,000 ×g for 5 min. The levels of Calcein-AM release to the supernatant were measured using a microplate spectrofluorimeter at emission of 527 nm under excitation at 485 nm. The TRAMP-C2 cell cultures in the absence of effector cells were used as background or spontaneous release controls, or those containing 2% Triton X-100 were used as maximum release controls. The cytotoxicity of macrophages or splenocytes was presented as the percentage of Calcein-AM released from target TRAMP-C2 cells into the media compared to the total Calcein-AM dye in the Triton X-100 treated cells and was calculated as follows: cytotoxicity (%) = [(test release − spontaneous release)/(maximum release − spontaneous release) × 100.

### 2.9. Animal Model of PCa: C57BL/6 TRAMP Mice

In this study, mouse breeding and all the experiments using mice were performed in accordance with the Canadian Council on Animal Care guideline under the protocols approved by the Animal Use Subcommittee at the University of British Columbia (Vancouver, BC, Canada).

TRAMP mice in C57BL/6 strain background for the ARR_2_BP (rat probasin derived gene promoter)-SV40Tag transgene (C57BL/6 TRAMP mice) were originally purchased from Jackson Laboratories (Bar Harbor, ME, USA) [21] and were maintained and housed in the Animal Care Facility of Jack Bell Research Centre (Vancouver, BC, Canada). The heterozygous transgenic male F1 of female TRAMP C57BL/6 × male nontransgenic C57BL/6 mice were used for the experiments. PCR-based screening of the transgenes in these mice was performed in genomic DNA samples using PCR primer pairs as described previously [21, 30]. PCa in male TRAMP mice has been characterized in previous studies as follows: mild-to-severe hyperplasia at age of 8–12 weeks; severe hyperplasia and adenocarcinoma at 18 weeks; and poorly differentiated and invasive adenocarcinoma at 24–30 weeks [31].

### 2.10. Experimental Groups and Treatment With DI Product

Three experimental groups were included in this study: onset (*n* = 6), control (*n* = 12), and DI extract-treated (denoted as extract, *n* = 18). Onset group were the mice at the age of 18 ± 1 weeks (before being assigned to any treatment). In control group, mice at 18 ± 1 weeks old were assigned to receive plain drinking water for the next 3 months. In extract group, when mice were 18 ± 1 weeks old, they were fed with dried DI product that was dissolved in drinking water at dose of 0.5 mg/mL. This dose did not affect drinking behavior of mice in our pilot study and gave the dosage of approximately 67 mg/kg/day of extract treatment as calculated based on the average water intake per an adult mouse (average body weight: 30 g) per day of approximately 4 mL [67 mg/kg/day = (0.5 mg/mL × 4 mL)/0.03 kg]. The mice in extract group received the herbal DI extract-containing drinking water for 3 months as the mice in control group. All the mice in these three groups were kept in a conventional room of the animal care facility before euthanasia (Jack Bell Research Centre, Vancouver, BC, Canada).

### 2.11. Histological Grading of PCa

In onset group, mouse prostate gland was harvested and weighted without further histological analysis. In both control and extract groups at the end of three months of treatment, the whole prostate gland from each mouse was collected, weighted, and gently washed with cold PBS. The tissue was fixed in 10% formalin and embedded in paraffin. The section was stained with Hematoxylin and Eosin (H&E) for histological grading of PCa. The disease grading of each sample was performed according to the pathological characters of prostatic intraepithelial neoplasia (PIN) and adenocarcinoma as previously described [32] in a blinded fashion as follows: (1) normal prostate; (2) early intraepithelial neoplasia indicated by “tufting up” of the epithelial layer, similar to a low grade PIN lesion in humans; (3) advanced intraepithelial neoplasia evidenced by extensive infolding of epithelial layers into the lumen, similar to a more advanced PIN lesion in humans; (4) well-differentiated prostate adenocarcinoma presented by early penetration of the glandular basement membrane by tumor cells that extend into the stromal compartment; (5) moderately differentiated prostate adenocarcinoma characterized by tumor formation of primitive glands lacking an obvious lumen; (6) poorly differentiated adenocarcinoma, the most severe grade of PCa shown by tumors composed of sheets and cords of highly pleomorphic anaplastic tumor cells. The pathological change was scored in a total of ten nonoverlapping microscopic views in two separate tissue sections and was presented as an average of score numbers for each mouse.

### 2.12. Determination of Splenocyte Phenotypes

A single cell suspension of splenocytes was prepared by gently crushing the spleens of mice in phosphate-buffered saline (PBS) in a cell strainer (BD-Canada, Mississauga, ON, Canada), followed by removal of erythrocytes by a brief incubation (~4 min) with lysis buffer (0.15 M NH_4_Cl, 1.0 mM KHCO_3_, 0.1 mM EDTA, pH 6.8). The phenotypes (CD4^+^, CD8^+^, CD45R/B220^+^, NK (pan)^+^, CD11b^+^, and Mac3^+^ cells) of the splenocyte population were determined by fluorescence-activated cell sorting (FACS) analysis using fluorescent-labeled rat monoclonal antibodies [anti-CD4 (clone YTS191-1), anti-CD8A (clone 53-6-7), anti-CD45R/B220 (clone RA3-6B2), anti-NK (pan) (clone DX5), anti-CD11b (clone M1-70-15), and anti-Mac3 (clone M3-84)] antibodies following the manufacturer's protocol (eBioscience, San Diego, CA, USA). Briefly, cells were probed with each fluorescence dye-conjugated antibody in the dark for 30 min at 4°C. After washing with PBS, the positively stained cells were detected by using flow cytometry and further quantified using FlowJo software (Tree Star Inc.).

### 2.13. Statistical Analysis

Data were presented as either mean ± standard derivation (SD) or mean ± standard error of the mean (SEM). Analysis of variance (ANOVA) or two-tailed *t*-test (nonparametric) was performed as appropriate for comparing the data between groups. A *P* value of ≤0.05 was considered significant.

## 3. Results

### 3.1. Composition of DI Product

Data (average of 3 separate measurements) from the chemical composition analysis of DI using spectrophotometric methods showed that the DI extract contained 15.95% (w/w) of total sugar including polysaccharides, 29.85% (w/w) of triterpenoids saponins, 0.15% (w/w) of flavonoids, and 1.15% (w/w) of protein, suggesting that the major components of this product were triterpenoids and polysaccharides/sugar.

**Figure 1:**
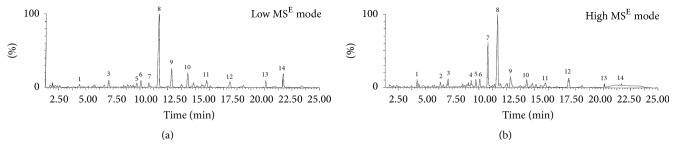
Typical UPLC-Qtop MS BPI chromatograms (ESI^+^ ionization) of DI product. (a) Low MS^E^ mode, 1.30e6. (b) High MS^E^ mode, 2.60e5.

The chemical substances of triterpenoids, flavonoids, and others in this product were further analyzed by using UPLC-Qtof-MS system, followed by a fragment match with the database in the “Chinese Medicine” library at Shenzhen Research Institute of the Hong Kong Polytechnic University (Shenzhen, Guangdong, China). The experiment on ESI^−^ ionization modes (data not shown) did not show as many peaks as on ESI^+^, and, by ESI^+^, a total of 14 ion peaks were clearly identified (Figure 1), and these ion peaks were matched to the chemical substances (i.e., ganoderic acid, glycyrrhizin, Wuweizi alcohol, aschantin, and schisandrin) mainly from* Glycyrrhiza *spp.,* Ganoderma lucidum*, and* Schisandra chinensis* (Table 2).

### 3.2. Activation of Macrophages by DI Product In Vitro

**Figure 2:**
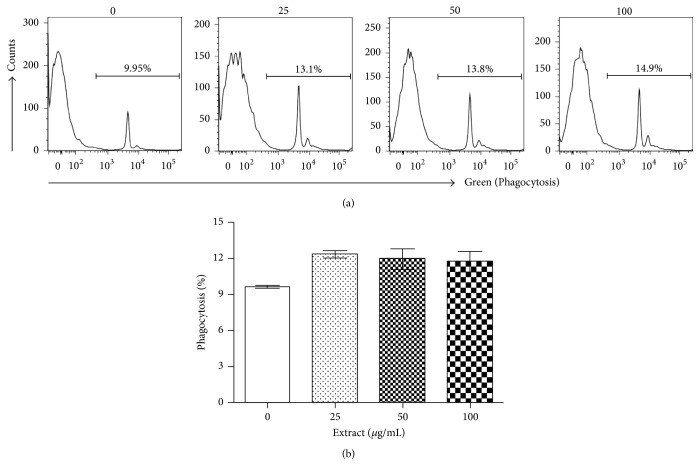
The DI activates macrophages (RAW264.7 cells). RAW264.7 cells are mouse macrophages cells and were incubated with different concentrations of herbal extract (0, 25, 50, or 100 *μ*g/mL) for 24 h. The phagocytosis was determined by engulfing FITC conjugated beads, and was quantitated as the percentage of FITC-positive cells by using a flow cytometry. (a) A typical flow cytometric graph showing the amount of engulfed beads inside the cells in each treatment. (b) The data were presented as mean ± standard derivation (SD) of each treatment (*P* = 0.0288 compared to “0” control, one-way ANOVA, *n* = 6).

The activation of anti-tumor-cytotoxic activity of macrophages has been considered an effective immunotherapy against tumor progression [33], and activation of phagocytosis is one of the tumor killing mechanisms by macrophages [34]. Thus, we first tested if the DI product (25–100 *μ*g/mL) was able to activate the phagocytosis of macrophages. As shown in Figure 2, the DI product at all tested concentrations stimulated phagocytosis of cultured RAW264.7 cells as compared to that in untreated cultures (*P* = 0.0288, one-way ANOVA, *n* = 6), and 25 *μ*g/mL resulted in the highest level of phagocytosis (12.32 ± 0.75) as compared to 9.64 ± 0.3 in untreated controls (*P* < 0.0001, two-tailed *t*-test, *n* = 6).

**Figure 3:**
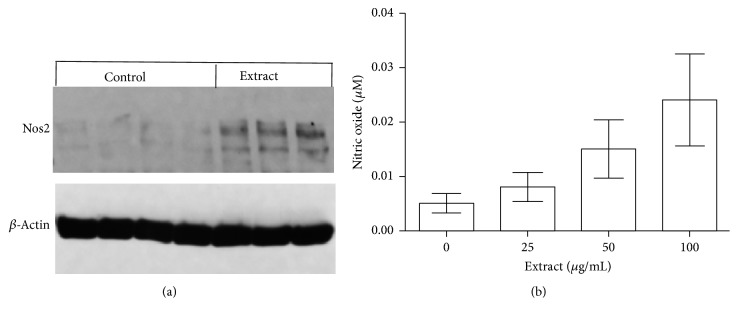
The DI stimulates nitric oxide (NO) production by upregulation of NOS2 expression in macrophages. Protein was extracted from cultured RAW264.7 macrophages after 24 h of stimulation with 100 *μ*g/mL of herbal extract (*n* = 3) or medium only (Control, *n* = 4). (a) Western blot analysis of NOS2 protein expression. (b) NO production in RAW264.7 macrophage cultures stimulated with different concentrations of herbal extract (0, 25, 50, or 100 *μ*g/mL) for 24 h. The data were presented as mean ± SD of each treatment (*P* = 0.0284 compared to “0” control, one-way ANOVA, *n* = 7). NO production (over 10 *μ*M) in LPS-stimulated cultures was not included.

To investigate the pathways by which the DI product-activated macrophages, the expression of a panel of 84 inflammation/immune response-related genes was examined in 100 *μ*g/mL herbal extract-stimulated RAW264.7 cells compared to untreated control (Suppl Table 1). Among these genes, eight genes* (c4b, cxcl3, kng1, lta, Nos2, Tlr1, Tnf, and Tnfsf14)* were significantly co-upregulated and only one* (il23a)* was downregulated (Table 3). The upregulation of Nos2 transcript by the DI product in cultured macrophages was further confirmed by Western blot analysis, showing an increase in expression of NOS2 protein (Figure 3(a)), as well as by a dose-dependent increase in NO production in cultures, from 0.005 ± 0.005 *μ*M in untreated controls to 0.024 ± 0.019 *μ*M in cultures treated with 100 *μ*g/mL of herbal extract (*P* = 0.0284, one-way ANOVA, *n* = 7) (Figure 3(b)).

### 3.3. Stimulation of Tumor Killing of Immune Cells by DI Product In Vitro

**Figure 4:**
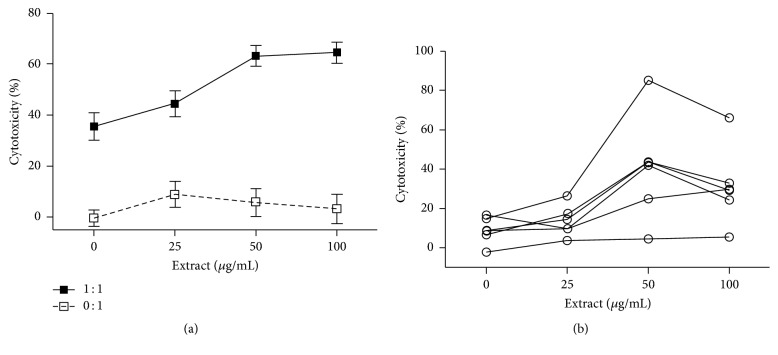
The DI activates the tumor-killing activity in cocultures of tumor cells with immune cells (macrophages or splenocytes). (a) RAW264.7 cells were cocultured with mouse prostate tumor cells (TRAMP-C2) (1 : 1, RAW264.7 : TRAMP-C2 with extract ratio) or without target cells (0 : 1, TRAMP-C2 with extract only). The data were presented as mean ± SD of each treatment (*P* < 0.0001 compared to “0” control, one-way ANOVA, *n* = 17–20). (b) The splenocytes from different tumor-bearing TRAMP mice (20–35 weeks old) were cocultured with mouse prostate tumor cells (TRAMP-C2) (1 : 1 ratio). The data were presented as mean of 6 determinants at each treatment for each mouse (*P* = 0.0152 compared to “0” control group, one-way ANOVA, *n* = 6). The cultures were stimulated with herbal extract at different concentrations (0, 25, 50, or 100 *μ*g/mL) for 24 h. The cytotoxicity was determined by the percentage of Calcein-AM release in Calcein-AM release assay.

To evaluate the antitumor activity of the DI product-activated immune cells, a cytotoxicity assay using cocultures was performed. As shown in Figure 4(a), without effector macrophages, the DI product alone did not have any significant cytotoxicity against TRAMP-C2 cells, whereas in cocultures with RAW264.7 cells, addition of the extract positively correlated with enhanced cytotoxicity against the tumor cells, indicated by the increase from 35.53 ± 5.3 in cocultures in the absence of the extract to 64.5 ± 4.17 in those stimulated by 100 *μ*g/mL of the extract (*P* < 0.0001, one-way ANOVA, *n* = 17–20). The activation of antitumor immune response by the DI product was further confirmed in TRAMP splenocytes. As shown in Figure 4(b), different levels of the extract-stimulated tumor killing of splenocytes were seen from different TRAMP mice, but were increased in a dose-dependent manner. Statistical analysis of the accumulating data from all of these 6 mice showed that the cytotoxicity was 8.96 ± 2.75 in cocultures without extract treatment, followed by an increase to 13.49 ± 3.19 in cocultures treated with 25 *μ*g/mL of extract and an increase to 40.65 ± 10.9 with 50 *μ*g/mL and 31.27 ± 7.99 with 100 *μ*g/mL (*P* = 0.0152, one-way ANOVA, *n* = 6). Taken together, this data demonstrates a stimulation of anti-PCa cytotoxity of immune cells, including macrophages, by the DI product in cocultures.

### 3.4. Suppression of Tumor Progression in TRAMP Mice by Oral Administration of the DI Product

**Figure 5:**
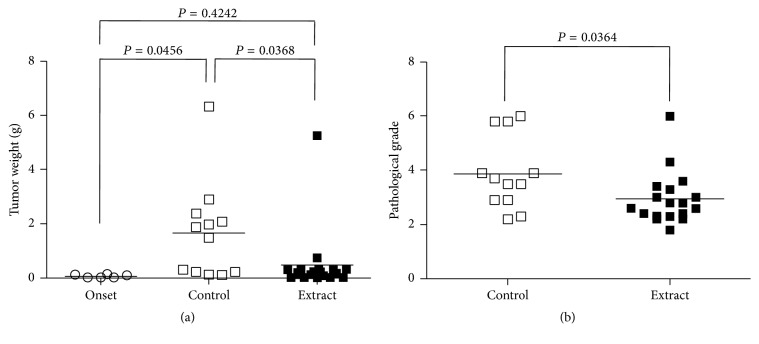
Prostate tumor weight and pathological grade. (a) The tumor weight from each mouse was weighted after euthanasia. (b) The pathological grading of prostate tumor in each mouse from control compared to herbal extract treatment. Onset: prior to any treatment; control: at the end of 3 months of plain drinking water; extract: at the end of 3 months of drinking water containing 0.5 mg/mL of the DI. Data were compared by using two-tailed *t*-test.

The efficacy of* in vivo* immune stimulation by the DI product against PCa development was tested in male TRAMP mice. After 12 weeks of treatment, as shown in Figure 5(a), the tumor weight was increased from 0.07617 ± 0.02313 g (*n* = 6) in mice before or at the beginning of treatment (Onset) to 1.671 ± 0.5124 g (*n* = 12) without any treatment (Control) (*P* = 0.0456, two-tailed *t*-test), but tumors did not grow in mice receiving the herbal extract in their drinking water, which is indicated by the fact that the tumor weight (0.4823 ± 0.2836 g, *n* = 18) in extract-treated mice was not significantly different from that in the “onset” group (*P* = 0.4242, two-tailed *t*-test), or the tumor growth was significantly suppressed as compared to those in control or untreated group (*P* = 0.0368, two-tailed *t*-test).

Tumor differentiation of PCa in these mice was graded by histology at the end of treatment.  Figure 5(b) indicated that, in consistent with the tumor growth suppression in the extract-treated group, the histological score of tumor grade (2.944 ± 0.2291, *n* = 18) was significantly lower than 3.887 ± 0.3836 (*n* = 12) observed in the control group (*P* = 0.0364, two-tailed *t*-test). We did not have histological data of the tumors from the “onset” group. Taken together, oral administration of the herbal extract significantly suppressed PCa development in TRAMP mice.

### 3.5. Stimulated Cytotoxicity of Splenocytes in TRAMP Mice by DI Product Treatment

To verify if the tumor suppression by DI product treatment was associated with an increase in anti-tumor immunity in mice, the proportion of effector immune cells in the spleens and their tumor-killing activity were examined. As indicated in Table 4, the percentage of CD8 T cells (CD8^+^), NK cells (NK1.1^+^), macrophages (Mac-3^+^), and dendritic cells (DC, CD11b^+^) decreased in mice after 3 months of tumor growth in control group as compared with that in “onset” group, and these decreases were not seen in mice receiving the DI product as no difference in the percentage of these effector cells was seen between onset and extract-treated groups. These data suggested that daily intake of DI product specifically maintained these effector immune cells systemically.

**Figure 6:**
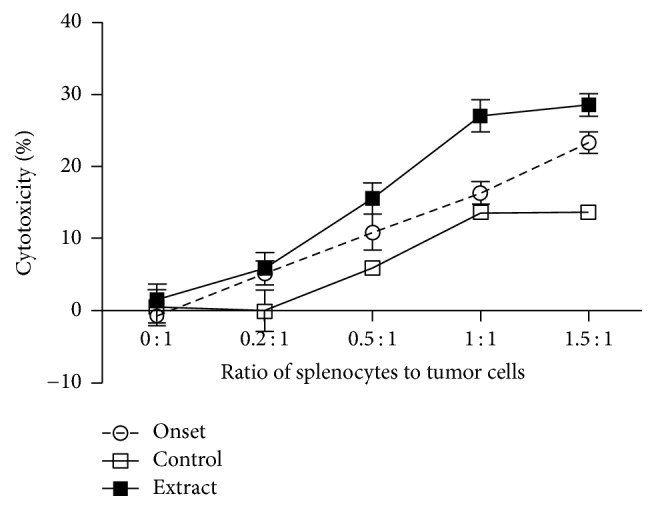
The tumor-killing activity of splenocytes from mice after treatment with the DI. The splenocytes from TRAMP mice (pretreated/onset, untreated control, or extract-treated) as indicated in Table 2 were cocultured with mouse prostate tumor cells (TRAMP-C2) at different ratios for 24 h, and the tumor-killing activity was determined by the percentage of Calcein-AM release in Calcein-AM release assay. The data were presented as mean of the standard error of mean (SEM) of each group (control versus onset, *P* = 0.0005; extract versus onset, *P* = 0.0012; extract versus control, *P* < 0.0001, two-way ANOVA, *n* = 6–12).

The tumor-killing activity of these splenocytes was further examined in cocultures with TRAMP-C2 cells at different ratios. As shown in Figure 6, the cytotoxic activity of splenocytes in the lysis of PCa cells in control group was lower than that in the “onset” group (*P* = 0.0005, two-way ANOVA, *n* = 6), and herbal extract treatment significantly stimulated splenocyte cytoxicity against tumor cells (*P* = 0.0012, extract versus onset; *P* < 0.0001, extract versus control, two-way ANOVA), indicating that the antitumor immune activity of these mice could be ranked in the order of high to low as follows: extract-treated > onset > untreated control.

## 4. Discussion

Cancer immunotherapy is a therapeutic approach by which natural antitumor immunity is activated or restored and has been approved for the treatment of many types of cancer, including melanoma, squamous cell lung cancer, and metastatic renal cell carcinoma [35], and it is also a recently emerging therapy for controlling metastatic castration-resistant PCa [36]. There are various specific and nonspecific agents that either suppress or stimulate the immune response. These include cytokines, cytokine receptors, therapeutic antibodies, and drugs [37], but increasing evidence in the literature suggests that immunodrugs that target a single pathway or molecule are of limited value, especially in cancer immunotherapy [38, 39]. It has been suggested that the activation of multiple immune responses by using “cocktails” of agents that simultaneously or synergistically stimulate many different vital components of the immune systems is required to develop the most efficacious immunotherapy [38, 39]. Botanicals produce a diverse range of natural products with immunomodulating potential against cancer [40], including polysaccharides from* G. lucidum* [41, 42], and triterpenoid saponins, such as astragalus saponin and astragaloside IV from* A. membranaceus* [43, 44]. These studies suggest that the immunostimulant botanicals could be used in the immunotherapy for the treatment of human cancer.

The DI product is a polyherbal formulation prepared by extraction of eight different Chinese medicinal herbs (*A. membranaceus, C. pilosula, G. lucidum, E. senticosus, L. lucidum, S. chinensis, A. macrocephala, *and* Glycyrrhiza *spp.) with ethanol and water (Table 1). Our chemical analyses show that the major components of this product are triterpenoid saponins (29.85%, w/w) and sugar including polysaccharides (15.95%, w/w). The saponins are mainly identified as glycyrrhizin or glycyrrhizic acid from* Glycyrrhiza *spp. and ganoderic acids and ganoderenic acid B from* G. lucidum* based on UPLC/Qtof-MS detection (Figure 1, Table 2). However, compounds from other plants were not identified by this assay, which might be due to less amounts of them in the final product and/or low sensitivity of the UPLC/Qtof-MS detection to these compounds. Ganoderic acids are the major components of saponins in* G. lucidum* [45], and glycyrrhizin is the major component of saponins in* Glycyrrhiza *spp. [46]. Furthermore, due to the limitation of our chemical analysis, this product may also contain small amounts of other triterpenoid saponins. The immunostimulant activities of polysaccharides isolated from* A. membranaceus*,* C. pilosula*,* G. lucidum*, and* Glycyrrhiza *spp. have been reported by many groups in mice [13, 47–49]. Polysaccharide treatment promotes antibody production and Th1 immune response and DC maturation [47–49] and also suppresses tumor growth by an increase in activation of B cells and macrophages, as well as reducing proinflammatory cytokine production [14]. Similar immunostimulation of triterpenoid saponins, particularly ganoderic acids and glycyrrhizin, has also been documented in the literature. For examples, treatment with ganoderic acid or glycyrrhizin suppresses tumor growth and increases NK cells activity in mice [50, 51]. In this study, we observed similar results, indicated by that addition of DI product containing both polysaccharides and saponins activated macrophages by upregulation of NOS2, TNF-alpha, and LT. The oral administration of this product restores the population of effector CD8 T cells, macrophages, and DCs in the spleens of PCa-bearing mice. This in turn is associated with increased antitumor cytotoxicity of splenocytes and prevention of PCa progression. All these studies may imply that the use of “cocktails” of immunostimulant polysaccharides and triterpenoid saponins may have the capacity to activate many different cell types (macrophages, DCs, T cells, B cells, and NK cells) of the immune system simultaneously. This may be superior to a single immunostimulant agent in the (re)activation of antitumor immunity. Furthermore, it was noticed that different dose-dependent responses to DI product were seen between phagocytosis activation (Figure 2(b)) and NO production (Figure 3(b)) in macrophages, suggesting that these responses (phagocytosis versus NO production) were activated by different substances in DI, and some of them might negatively regulate the phagocytosis that was especially seen in macrophages treated with higher concentrations of DI (>25 *μ*g/mL) (Figure 2(b)).* In vivo* oral feeding with DI suppressed PCa growth but not completely in all of mice (Figure 5(a)), implying that activation of antitumor immunity and/or other unknown cytotoxicity mechanisms by DI feeding could not completely block the pathways of tumor growth in this model.

Do we have a target of an immunotherapy or immunostimulatory strategy for controlling the progression of low-risk PCa in patients? By immunohistochemical methods, DCs (or Langerhans cells) and HLA class II-positive macrophages are mostly identified only in prostate carcinoma of a low grade (grades 1-2) [52]. Shimura et al. further demonstrate that, in prostate cancer specimens, CD68-positive macrophages are distributed in three distinguishable compartments (stroma, tumor cell region, and tumor-containing lumens), with 84% of macrophages being in the tumor-associated stroma and the remaining 16% in the tumor cell region and the lumens composed of tumor cells [53]. Similarly, stroma-infiltrating macrophages and compartment-specific macrophage densities are only found in PCa tissues with lower grade (Gleason score, ≤6) or with lower clinical stage [53]. It has been well documented that activated macrophages kill tumor cells by many mechanisms, such as antibody dependent-cell mediated cytotoxicity [54], phagocytosis [55], and protease secretion [56]. As well, tumor antigen presenting cells (the same as DCs) stimulate anti-tumor T cell immunity including cytotoxic CD8 T cells [57]. Therefore, the tumor-associated macrophages as well as DCs in low grade PCa could be a candidate for immunotherapy targets to eliminate slowly growing prostate tumor cells or to control the progression of prostate tumor development in the clinic.

In conclusion, data from this experimental study indicate that herbal products (like the DI product) containing different immunostimulant components and activating different immune cells, particularly macrophages or DCs offer promise as agents for secondary prevention of PCa from progression after PCa diagnosis, particularly for those patients with low-risk PCa and on AS. Each herb is added to this particular polyherbal formulation on the basis of its traditional uses. There are, however, limited data in the literature showing the immunoactive ingredients, their mechanisms of action, and their safety and efficacy in human subjects. Further studies of the efficacy of this herbal product or similar, modified products against PCa progression are required using a human preclinical model and in human subjects.

## Figures and Tables

**Table 1:** The herbal ingredients of the DI product (liquid)^*∗*^.

List of herbs	Plant part	Extraction ratio	Quantity crude equivalent (mg/mL)
*Astragalus membranaceus*	Root	1 : 4	50
*Codonopsis pilosula*	Root	1 : 4	37.5
*Ganoderma lucidum*	Root	1 : 4	37.5
*Eleutherococcus senticosus*	Fruiting body	1 : 3	50
*Ligustrum lucidum*	Fruit	1 : 4	25
*Schisandra chinensis*	Fruit	1 : 4	25
*Atractylodes macrocephala*	Rhizome	1 : 4	25
*Glycyrrhiza *spp.	Root and stolon	1 : 5	10

^*∗*^Provided by the St. Francis Herbal Farm Inc.

**Table 2:** Chemical substances identified by UPLC-Qtop MS, followed by the match with the Traditional Medicine Library provided within the UNIFI informatics platform.

Peak number	*m*/*z*	Chemical name	Mass error (ppm)	Source(s)
1	257.0805	Liquiritigenin	−1.4	*Glycyrrhiza *spp.
2	257.0809	Isoliquiritigenin	0.06	*Glycyrrhiza *spp.
3	285.0756	3′-Methoxydaidzein	−0.64	*Glycyrrhiza *spp.
4	515.3002	Ganoderic acid C, D, LM2, *ς*	−0.32	*G. lucidum*
		Ganoderenic acid A, B		
5	269.0807	Formononetin	−0.45	*Glycyrrhiza *spp.
				*A. membranaceus*
6	573.3056	Ganoderic acid H	−0.3	*G. lucidum*
7	823.4117	Glycyrrhizin	0.83	*G. lucidum*
8	433.2223	Wuweizi alcohol	0.51	*S. chinensis*
9	401.1598	Aschantin	0.86	*S. chinensis*
10	401.1956	Schisandrin B	−0.65	*S. chinensis*
11	415.1748	Cniforin B/coumarin CC3	−0.77	*Glycyrrhiza *spp.
12	417.227	Schisandrin A (deoxyschizandrin)	−0.44	*S. chinensis*
13	401.1955	Ergosterol	0.63	*G. lucidum*
14	431.3517	Ergosterol peroxide	0.26	*G. lucidum*

These chemicals belong to five different groups: triterpenoids (ganoderic acids, ganoderenic acids, and glycyrrhizin); flavonoids (liquiritigenin, isoliquiritigenin, 3-methoxylaidzein, and formononetin); phenolics/ligans (wuweizi alcohol, aschantin, and schisandrins); sterols (ergosterol, ergosterol peroxide); and coumarins (cniforin B).

**Table 3:** Induction of gene expression in cultured RAW264.7 macrophages.

Gene name	Functions	Fold difference (treated versus untreated)	*P* value (*t*-test, *n* = 3)
C4b	Complement component	2.69	0.0155
*Cxcl3*	Chemokine ligand 3, a chemoattractant for neutrophils	8.45	0.0006
*Il23a*	Interleukin 23 p19 for memory T cells	−1.56	0.0276
*Kng1*	Suppressing tumor cell proliferation	13.56	0.0331
*Lta (Tnfb)*	Lymphotoxin alpha, immunostimulation	2.89	0.0029
*Nos2*	Nitric oxide synthase 2, producing NO	9.71	0.0061
*Tlr1*	Toll-like receptor 1, recognizing pathogen-associated molecular patterns	2.55	0.0038
*Tnf*	Tumor necrosis factor: inducing cell death	2.89	0.0081
*Tnfsf14*	TNF (ligand)superfamily, member 14, stimulating T cell proliferation and inducing cell death	2.99	0.0108

RAW264.7 macrophages were stimulated in the absence (untreated, culture medium only) or presence of 100 *µ*g/mL of DI product in culture medium (treated) for 24 h. A panel of 84 genes related to “Inflammatory Response and Autoimmunity” was determined using PCR array. Data are presented as a list of gene expression that is statistically changed by extract treatment in four separate experiments.

**Table 4:** Phenotypes of splenocytes from TRAMP mice before (onset group) or after 3-month treatment (both control and extract groups).

Phenotypes (%)	Onset (*n* = 5)	Control (*n* = 5)	Extract (*n* = 12)	*P* value (onset versus control)	*P* value (onset versus extract)	*P* value (control versus extract)
CD4	18.82 ± 2.18	16.6 ± 2.89	17.18 ± 2.85	0.2080	0.2714	0.7074
CD8	14.11 ± 3.18	8.29 ± 0.88	11.6 ± 3.32	0.0043	0.1713	0.0474
CD45R/B220	49.14 ± 19.26	66.2 ± 3.17	58.36 ± 12.48	0.0864	0.2539	0.1929
NK1.1	3.74 ± 0.82	2.22 ± 0.05	3.21 ± 1.46	0.0031	0.4619	0.1549
Mac3	9.85 ± 2.31	2.4 ± 0.57	7.34 ± 5.92	0.0001	0.3787	0.0872
CD11b	6.9 ± 1.09	4.98 ± 0.94	7.44 ± 2.58	0.0177	0.6582	0.0579

Phenotypes of splenocytes (CD4: CD4^+^ T cells; CD8: CD8^+^ T cells; CD45R: B cells; NK1.1: NK cells; Mac3: macrophages; CD11b: DCs) were determined by FACS analysis. Data are presented as mean ± SEM in each group, and were compared using two-tailed *t*-test.

## Supplementary Materials

Supplementary Materials Expression of a panel of Inflammatory Response And Autoimmunity-related eighty-four genes in RAW264.7 macrophages after stimulation with Deep Immune product (Suppl Table 1). Suppl Table 1: Inflammatory Response And Autoimmunity-related gene expression in the Deep Immune-treated RAW264.7 macrophages compared to those in untreated controls.
