# Supplementary material for: Prevention of Prostate Tumor Development by Stimulation of Antitumor Immunity Using a Standardized Herbal Extract (Deep Immune®) in TRAMP Mice
Source: Evid Based Complement Alternat Med. 2018 May 14;2018:9707543. doi: 10.1155/2018/9707543 (PMC5976932; doi:10.1155/2018/9707543)
Supplement: Supplementary Materials — Expression of a panel of Inflammatory Response And Autoimmunity-related eighty-four genes in RAW264.7 macrophages after stimulation with Deep Immune product (Suppl Table 1). Suppl Table 1: Inflammatory Response And Autoimmunity-related gene expression in the Deep Immune-treated RAW264.7 macrophages compared to those in untreated controls. [file 9707543.f1.docx]

**Suppl TABLE 1:** Inflammatory Response and Autoimmunity-related gene expression in the Deep Immune^®^-treated RAW264.7 macrophages compared to those in untreated controls.

| Functions | Gene name | Fold change (treated vs. untreated) | *p* value (t-test, n = 3) |
| --- | --- | --- | --- |
| Cytokines (Chemokines) | *Ccl1* | 5.97 | 0.1534 |
|  | *Ccl11(Eotaxin)* | 1.1 | 0.6470 |
|  | *Ccl12* | 1.54 | 0.4546 |
|  | *Ccl17(Tarc)* | 1.68 | 0.0505 |
|  | *Ccl19* | 2.74 | 0.1650 |
|  | *Ccl2(Mcp-1)* | 1.45 | 0.5790 |
|  | *Ccl20* | 4.17 | 0.5534 |
|  | *Ccl22 (Mdc)* | 1.97 | 0.1173 |
|  | *Ccl24 (Eotaxin-2)* | -5.44 | 0.0522 |
|  | *Ccl25* | 1.13 | 0.6793 |
|  | *Ccl3 (Mip-1a)* | 1.56 | 0.3182 |
|  | *Ccl4 (Mip-1b)* | 1.72 | 0.4899 |
|  | *Ccl5 (Rantes)* | 2.32 | 0.0890 |
|  | *Ccl7 (Mcp-3)* | 1.09 | 0.5699 |
|  | *Ccl8 (Mcp-2)* | 1.97 | 0.3638 |
|  | *Cxcl1* | -1.97 | 0.3084 |
|  | *Cxcl10 (Inp 10)* | 1.58 | 0.3719 |
|  | *Cxcl11* | -1.71 | 0.8053 |
|  | *Cxcl2* | 3.49 | 0.1053 |
|  | *Cxcl3* | 8.45 | 0.0006 |
|  | *Cxcl5 (Ena78/Lix)* | 2.92 | 0.9547 |
|  | *Cxcl9 (Mig)* | 2.88 | 0.6747 |
| Cytokines (Interleukins and others) | *Il10* | 0.57 | 0.0750 |
|  | *Il17a* | 3.15 | 0.6061 |
|  | *Il18* | -1.02 | 0.9056 |
|  | *Il1a* | 2.59 | 0.3763 |
|  | *Il1b* | 3.22 | 0.1085 |
|  | *Il22* | 3.65 | 0.2037 |
|  | *Il23a* | -1.56 | 0.0275 |
|  | *Il5* | 2.02 | 0.3224 |
|  | *Il6* | 4.2 | 0.5750 |
|  | *Il7* | -1.05 | 0.6718 |
|  | *Il9* | -2.03 | 0.7867 |
|  | *Cd40Ig (Tnfsf5)* | 1.56 | 0.4339 |
|  | *Csf1 (MCSF)* | 1.79 | 0.1257 |
|  | *Fasl (Tnfsf6)* | -1.21 | 0.6169 |
|  | *Ifng* | 3.76 | 0.8347 |
|  | *Lta (Tnfb)* | 2.87 | 0.0029 |
|  | *Ltb* | 1.77 | 0.1108 |
|  | *Tnf* | 2.89 | 0.0081 |
|  | *Tnfsf14* | 2.99 | 0.0108 |
| Cytokine Receptors | *Il10rb* | 1.26 | 0.4231 |
|  | *Il1r1* | 4.5 | 0.5874 |
|  | *Il1rap* | 1.58 | 0.2322 |
|  | *Il1rn* | 1.77 | 0.5315 |
|  | *Il23r* | 4.35 | 0.3377 |
|  | *Il6ra* | 1.93 | 0.3029 |
| Chemokine receptors | *Ccr1* | 2.47 | 0.3105 |
|  | *Ccr2* | -1.05 | 0.9255 |
|  | *Ccr3* | 1.43 | 0.7081 |
|  | *Ccr4* | 1.78 | 0.3263 |
|  | *Ccr7* | 1.04 | 0.5292 |
|  | *Cxcr4* | -1.62 | 0.2566 |
|  | *Cxcr1 (Il8ra)* | -1.48 | 0.3249 |
|  | *Cxcr2 (Il8rb)* | 5.24 | 0.1334 |
| Cytokine metabolism | *Tir1* | 2.55 | 0.0038 |
|  | *Tir2* | 1.56 | 0.2210 |
|  | *Tir3* | 2.18 | 0.3561 |
|  | *Tir4* | 1.35 | 0.5118 |
|  | *Tir5* | 1.84 | 0.5641 |
|  | *Tir6* | 1.81 | 0.1655 |
|  | *Tir7* | 1.64 | 0.5987 |
|  | *Tir9* | 2.54 | 0.5216 |
| Cytokine-mediated signaling pathway | *Myd88* | 2.27 | 0.3175 |
|  | *Ripk2* | 2.35 | 0.0628 |
|  | *Tirap* | 2.39 | 0.0669 |
| Acute-phase response | *Ptgs2 (Cox2)* | 4.44 | 0.0554 |
| Regulation of inflammatory response | *Bcl6* | 3.07 | 0.2425 |
|  | *C3* | 1.21 | 0.5528 |
|  | *C3ar1* | 1.86 | 0.3865 |
|  | *C4b* | 2.69 | 0.0155 |
|  | *Cd40 (Tnfrsf5)* | 1.35 | 0.4339 |
|  | *Cebpb* | 1.28 | 0.6810 |
|  | *Crp* | -1.54 | 0.5448 |
|  | *Fos* | 2.1 | 0.2793 |
|  | *Itgb2* | 1.84 | 0.5890 |
|  | *Kng1* | 13.56 | 0.0331 |
|  | *Ly96 (MD-2)* | 1.12 | 0.6243 |
|  | *Nfkb1* | 2.17 | 0.1274 |
|  | *Nos2 (iNOS)* | 9.71 | 0.0061 |
|  | *Nr3c1 (Gr1)* | 1.7 | 0.4933 |
|  | *Sele* | 2.15 | 0.9678 |
|  | *Tollip* | 2.14 | 0.0529 |

The levels of “Inflammatory Response and Autoimmunity”-related genes were analyzed using mouse PCR array (Catalog No. PAMM-077Z, QIAGEN, Toronto, ON, Canada).
